# Procursive Epilepsy

**Published:** 1889-06-22

**Authors:** 


### Within the Wards.

### PROCURSIVE EPILEPSY.

Modern medicine is marked by an exceedingly careful and thorough study of diseased conditions, with their causes and concomitants; and this leads to accurate differentiation and description and to soundly scientific classification. What is called "procursive epilepsy" is hardly known, even by name, to the general public, and epilepsy itself is but little understood. Yet it is often of the very first importance both in the interests of public justice and of private conduct that the nature, consequences, and history of the epileptic condition be thoroughly familar to judge, jury, and all concerned. A child or a grown-up person suddenly, and without reason, takes to running forward in a line as straight as an arrow's flight from the place in which he stood. He runs as if he were "possessed," and indeed he is "possessed." He cannot help running. He is not conscious that he is running. If anything stand in his way, even a stone wall, he must run against it with all his might. If a precipice or a deep river be in front of him he must still run, and be dashed to pieces or drowned. The running may last as long as an ordinary epileptic fit. Strange to say, the patient seldom falls down, or becomes comatose as in ordinary epilepsy, but his face is very red and congested. That is one form of "procursive epilepsy." In other instances the patient, instead of running forward, "rotates," or goes round and round in large circles; and this performance may also continue as long as a common fit of epilepsy. The case of a boy of thirteen is reported, who before that age had not been subject to epileptic attacks at all. In a moment, without warning, or feeling, or cry of any kind, he started off running in a forward direction. He ran for a considerable distance, and then gradually "came to." There was neither fall nor foaming at the mouth, but the seizure was as distinct and definite as if all the usual accompaniments of epilepsy had been present. Soon the attacks were repeated daily, and afterwards several times a day. Under the continued influence of the fits, this poor boy became a totally different kind of person. His instincts were perverted, and after each seizure he was reduced for a time to a condition of hopeless stupidity. The epileptic condition is entirely due to abnormal processes in the brain, and is altogether beyond the control of the sufferer himself. Unhappily, also, the most carefully thought-out measures of scientific medicine, though they often relieve, seldom result in complete cure. The facts to be borne in mind by non-medical readers are these—that during the seizure, whatever an epileptic may do he is not responsible; that in the intervals, even when he seems quite well, he is much less responsible than ordinary persons; and that as a rule the tendency to bodily and mental imbecility is progressive. No class of persons is more deserving of sympathy and help than pronounced epileptics.

